# Outlier-Robust Three-Element Non-Uniform Linear Arrays Design Strategy for Direction of Arrival Estimation in MIMO Radar

**DOI:** 10.3390/s25165062

**Published:** 2025-08-14

**Authors:** Andrea Quirini, Fabiola Colone, Pierfrancesco Lombardo

**Affiliations:** Department of Information Engineering, Electronics and Telecommunications DIET, Sapienza University of Rome, 00184 Rome, Italy; fabiola.colone@uniroma1.it (F.C.); pierfrancesco.lombardo@uniroma1.it (P.L.)

**Keywords:** MIMO radar, AESA radar, non-uniform linear arrays, direction of arrival, maximum likelihood estimation

## Abstract

This paper presents a novel design strategy for outlier-robust, three-element non-uniform linear array (NULA) configurations optimized for multiple-input multiple-output (MIMO) radar systems aimed at target direction of arrival (DoA) estimation. The occurrence of outliers, i.e., ambiguous estimates, is a well-known issue in DoA estimation based on the maximum likelihood (ML), which is caused by the local maxima of the likelihood function. Specifically, we study how the positioning of both transmitters and receivers affects both presence of outliers and accuracy of ML DoA estimation. By leveraging a theoretical prediction of the DoA mean squared error (MSE), we propose a design strategy to jointly optimize the positions of NULA array of three transmitting and receiving elements, only inside a subspace which guarantees that the outlier probability remains below a specified threshold. Compared to NULA configurations with a single transmitter, the proposed designs achieve superior estimation accuracy due to two key factors: improved asymptotic performance resulting from a narrower mainlobe, and enhanced robustness against outliers due to reduced sidelobes. Furthermore, the proposed approach is well-suited for practical implementation in low-cost radars using only 3 × 3 or 2 × 3 MIMO configurations, as it also incorporates practical design constraints such as minimum inter-element spacing to account for the physical dimensions of the antennas, and tolerance in the installation accuracy.

## 1. Introduction

Active electronically scanned array (AESA) radar architectures are well-established in radar surveillance, due to their enhanced flexibility compared to conventional radar systems. By using multiple transceiver modules, AESA radar systems allow for electronic control of the antenna beam direction, enabling rapid and precise scanning of the surveilled area without the need for physical movement of the antenna.

In recent years, the fully digital paradigm for AESA radar has gained attention in both industry and research, as it leverages digital signal processing to further enhance the flexibility of radar operations. Specifically, fully digital AESA radars aim to provide independent digital control of each antenna element for both transmission and reception. This is achieved by moving analog-to-digital converters (ADCs) and digital-to-analog converters (DACs) as close as possible to the antennas, allowing most signal processing operations to be implemented in the digital domain.

Next-generation fully digital arrays are currently employed in only a limited number of radar products, typically operating in single transmit mode. However, the independent control of transmit and receive antennas facilitates multiple-input multiple-output (MIMO) operations. Specifically, in fully digital AESA radar systems, each antenna can transmit different orthogonal waveforms directly synthesized in the digital domain, ensuring MIMO functionality and enabling flexible and efficient exploitation of the available antenna aperture.

A major challenge in MIMO radar systems is ensuring the orthogonality of waveforms, particularly when multiple transmit antennas are involved. Achieving orthogonality across a large number of waveforms over a sufficiently wide range and across the Doppler frequencies of interest is a nontrivial problem. Chirp signals, particularly linear frequency-modulated (LFM) signals, are known to offer quasi-orthogonal waveform pairs, such as the “chirp up” and “chirp down” pair. Additionally, further quasi-orthogonal waveforms can be designed by utilizing “V-chirp” signals with bidirectional sweeps.

In low-cost radar systems for surveillance, there is a strong interest in leveraging the potential of MIMO operation without significantly increasing system complexity. This requires the following:

(A) The use of a limited number of antennas to reduce the number of receiving chains and the volume of data to be processed.
(B) The use of a few simple orthogonal or quasi-orthogonal waveforms, such as chirps, which do not require complex optimization to ensure orthogonality.

In this perspective, a radar system with an extremely limited number of channels can be interesting for many low-cost surveillance applications, as it reduces costs and requires using a limited number of orthogonal waveforms. Systems with a few antennas are also beneficial for designing lightweight and compact systems, suitable for installation on moving platforms such as vehicles or aircraft, where the capability to process large amounts of data is also limited.

When aiming to use a limited number of antennas, non-uniform linear array (NULA) configurations are ideal. These configurations allow for the strategic positioning of the available sensors, including both short and long baselines with a small number of elements, therefore optimizing the trade-off between performance and cost. Specifically, this paper focuses on the application of target direction of arrival (DoA) maximum likelihood (ML) estimation, considering a MIMO radar system with three antennas. Three-element array configurations are widely used for the practical and cost-effective implementation of radar systems for DoA estimation (e.g., see [1,2,3,4,5,6,7]). This choice is driven by the target applications of our design strategy: low-cost, resource-constrained systems such as automotive radar or sensors for small unmanned aerial vehicles, where size, weight, power, and computational load are critical constraints. Each antenna can be either a transmitting/receiving element or a receiving-only element. Our goal is to optimize the positions of the three antennas to maximize the estimation accuracy, while constraining the probability of outliers among the obtained DoA estimates.

Achieving accurate DoA estimation with such a limited number of antenna elements is especially challenging. Specifically, using a typical uniform linear array (ULA) configuration with only three antennas would be highly constraining, leaving significant performance potential unexplored. In contrast, NULA configurations can be quite beneficial when there are severe constraints on the number of antenna elements, as they enable more flexible designs that can better exploit the limited resources available.

However, compared to ULA configurations, it is well known that NULA configurations introduce DoA estimation ambiguities in the direction of one of the sidelobes of the likelihood function. These ambiguities can significantly degrade DoA estimation accuracy, causing the DoA estimation mean square error (MSE) to deviate from the asymptotic performance, especially under limited signal-to-noise ratio (SNR) conditions. The accuracy of DoA estimation in the presence of outliers has been extensively studied in the open literature [8,9,10,11,12,13,14,15,16,17], and several lower bounds for the MSE have been derived, including the Barankin bound [10], the Bayesian Cramér–Rao Bound (CRB) [11,12,13], and the Ziv–Zakai bound [14,15].

The problem of DoA estimation has been extensively addressed in the literature, with powerful super-resolution algorithms like MUSIC [18], ESPRIT [19], and methods based on compressed sensing [20,21,22] as the classical choices. These super-resolution techniques allow resolving multiple closely-spaced targets. However, the work presented here focuses on a different challenge, predominant in low-cost, resource-constrained systems: ensuring robustness against estimation outliers for a single target at low SNR. For this purpose, the ML estimator is particularly suitable, as its performance is well-understood theoretically [16], allowing establishment of a direct relationship between array geometry and outlier probability, which is the core of our contribution.

Notably, Ref. [16] presented an accurate theoretical prediction for the DoA MSE in moderate to high SNR scenarios. Therein, it is shown that the probability of outliers can be expressed only in terms of the operative SNR and of the array geometry. This highlights the importance of the array geometry, which should be carefully designed to maximize the robustness against outliers, especially under limited SNR conditions. Several efforts have been undertaken for designing ambiguity-resistant NULA configurations over the years [23,24,25,26,27,28,29], including minimum redundancy arrays (MRA) [23] and ambiguity-resistant interferometers [24]. Particularly, in our previous work [29], we built upon the MSE theoretical prediction in [16] to devise a flexible design strategy for outlier-robust three-element NULA configurations on receive. The design strategy proposed therein was only applicable to SIMO radar systems, allowing us to control the probability of outliers for estimating the DoA of either emitting sources, targets illuminated by existing transmitters of opportunity (as in passive radar), or in scenarios where only one of the three antennas was equipped with a transmitter.

In this paper, we extend this outlier-robust design framework to co-located MIMO setups, which represents the main contribution of this work. This extension is non-trivial since the physical position of each antenna element simultaneously affects multiple virtual channels. Through theoretical derivation, we demonstrate that for the same physical placement of antennas, a MIMO configuration with multiple transmitters emitting orthogonal waveforms yields a different virtual array geometry, which generally results in an increased robustness against outliers. In turn, this modifies the array design strategy, enabling the design of longer-baseline NULAs that offer superior asymptotic accuracy for the same level of outlier robustness guaranteed by shorter configurations in the SIMO case.

Based on this analysis, we propose a NULA design strategy that allows system designers to fully exploit the limited number of available antennas, achieving an optimal balance between asymptotic accuracy and outlier robustness by leveraging the advantages of MIMO operation. In the paper, we assume independent digital control over each antenna element, as guaranteed by next-generation AESA systems, and orthogonality between waveforms—an assumption that can be approximately satisfied easily in practice with a system using a maximum of three transmitters, e.g., using quasi-orthogonal up, down, and V-shaped LFM waveforms.

This paper is organized as follows. Section 2 presents the MIMO signal model and revisits the MSE theoretical approximation derived in [16]. Section 3 presents the proposed NULA design strategy for MIMO radar, based on a constraint on the maximum outlier probability. Section 4 provides an example of an application of the proposed design strategy, considering practical technological constraints such as minimum array inter-element spacings or installation tolerance. Lastly, Section 5 draws our concluding remarks.

## 2. Array Signal Model and MSE Prediction

**Figure 1:**
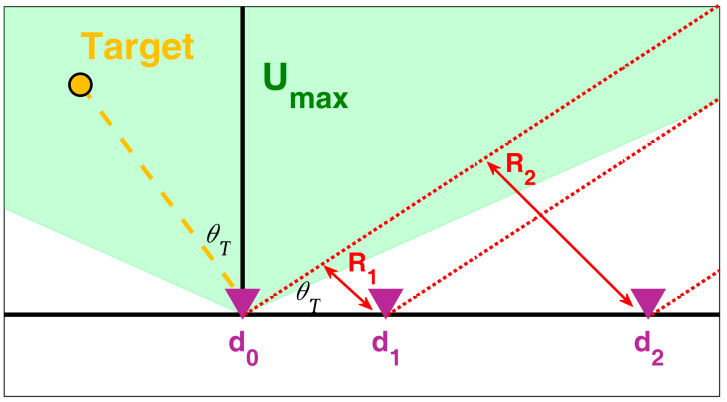
Array geometry and operative scenario.

Consider the system geometry depicted in Figure 1, showing a NULA with N elements aligned along the x-axis. Let $d_{n}$ denote the position of the n-th element. Without loss of generality, we assume that the leftmost element is at the origin of the axis, so that the array geometry is described by the vector $z=\left[d_{0},\;{\;d}_{1},\ldots ,d_{N-1}\right]$ collecting the positions of the array elements. Here, we assumed that the two-way phase center of the array element at the origin of the axis is the phase reference.

We consider a target T located within the surveilled area at an angle $\theta_{T}$ relative to the array boresight. Our signal model focuses on an isolated target, assuming it has been resolved within a specific range resolution cell. The array comprises M≤N transmitting elements, each emitting a waveform denoted as $s_{m}\left[k\right]$. The signal contribution at the n-th antenna after matched filtering to the m-th waveform and at the target range bin can be expressed as:

(1) $$x_{m,n}=A_{m,n}e^{-j2\pi \frac{d_{m}^{TX}}{\lambda }u_{T}}e^{-j2\pi \frac{d_{n}}{\lambda }u_{T}}+\sum_{i=0,i\neq m}^{M-1}{A_{i,n}\rho }_{m,\;i}e^{-j2\pi \frac{d_{i}^{TX}}{\lambda }u_{T}}e^{-j2\pi \frac{d_{n}}{\lambda }u_{T}}+w_{m,\;n},$$

where

- $A_{m,n}$ represents the complex target signal amplitude after range compression;
- λ is the signal wavelength;
- $u_{T}≜\sin\theta_{T}$ is the target DoA;
- $\left\{d_{m}^{TX},\;m=0,\ldots M-1\right\}$ is a subset of $\left\{d_{n},\;n=0,\cdots ,N-1\right\}$;
- $\rho_{m,n}$ is the complex amplitude of the cross-correlation interference, which models the leakage from the i-th transmitted waveform into the m-th processed channel.
- $w_{m,n}$ is a complex-valued sequence representing the thermal noise contribution at the n-th receiving element;

However, for LFM waveforms with a high time-bandwidth product, such as those typically used in radar, the energy of the cross-talk term is significantly lower than the peak energy of the auto-correlation term. For this reason, its effect can be reasonably modeled as a small degradation of the effective SNR. Therefore, the theoretical derivations are obtained assuming perfect orthogonality, but the impact of this non-ideal behavior will be considered in simulated examples later in the paper.

**Figure 2:**
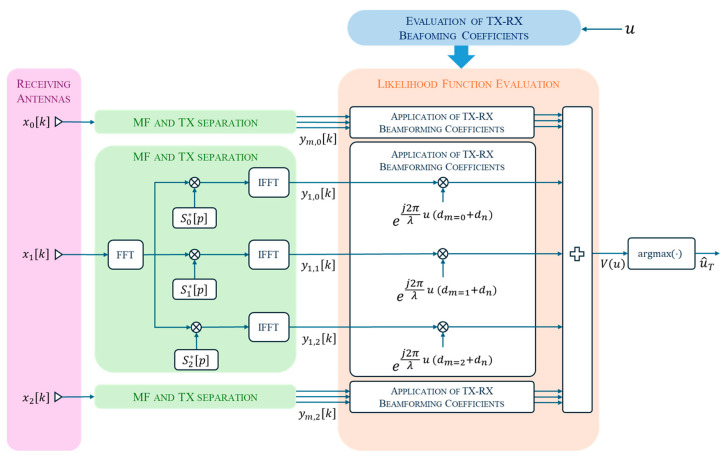
Block diagram of the MIMO processing scheme.

To obtain an estimate ${\hat{u}}_{T}$ of the target DoA $u_{T}$, we resort to the ML estimation technique. A block diagram of the fundamental processing scheme considered in this paper is shown in Figure 2. Specifically, the received signals at different antennas are first range-compressed via matched filtering, which involves multiplying the received signal spectrum $X_{n}\left[p\right]$ by the complex conjugate of the spectrum of each transmitted signal, $S_{m}\left[p\right]$. This is performed by the block named “MF and TX separation”, which is exploded in its details only for channel k = 1 for compactness. This operation enables both target range estimation and separation of the contributions from each transmitter at each receiver. For simplicity, in Figure 2, the range compression process is represented assuming that M=3 transmitters are present. In cases with M=1 or M=2 transmitters, the processing scheme is simplified accordingly.

To carry out a fair comparison of SIMO and MIMO, we assume that the total transmitted power is the same for every setup. Therefore, with M transmitters, the amplitudes $A_{m,n}$ are scaled by $\sqrt{M}$, to account for the power distribution across the array.

Following the processing scheme in Figure 2, each range-compressed sequence $y_{m,n}\left[k\right]$ undergoes a beamforming stage, where the transmit and receive phase shifts are compensated to estimate the target DoA. To this end, Equation (1) can be conveniently rewritten in vector notation as follows:

(2) $$x=A⊙s\left(u_{T}\right)+w,$$

where

- $x={\left[x_{1,1},\ldots ,x_{1,N},x_{2,1},\ldots x_{2,N},\ldots x_{M,1}\ldots ,x_{M,N}\right]}^{T}$ is a column vector of size $\left(NM\;\times 1\right)$, collecting the signal contributions $x_{m,n}$;
- $A={\left[A_{1,1},\ldots ,A_{1,N},A_{2,1},\ldots A_{2,N},\ldots A_{M,1}\ldots ,A_{M,N}\right]}^{T}$ is a column vector of size $\left(NM\;\times 1\right)$, collecting the complex amplitudes $A_{m,n}$;
- $s\left(\theta_{T}\right)$ is a steering vector of size $\left(NM\;\times 1\right)$, collecting the phase shifts $e^{-j2\pi \frac{d_{m}^{TX}+d_{n}}{\lambda }u_{T}}$ due to the two-way path;
- $w=CN\left(0,\sigma_{n}^{2}\right)$ is a column vector of size $\left(NM\;\times 1\right)$, collecting the MN thermal noise samples, drawn from a complex Gaussian distribution with zero mean and $\sigma_{n}^{2}$;
- ⊙ denotes the elementwise Hadamard product.

Based on this signal model, we can define an ideal likelihood function for our ML estimator as in [11]:

(3) $$V\left(u\right)={\left|s^{H}\left(u\right)x\right|}^{2},$$

Here, $s^{H}\left(u\right)$ represents the steering vector corresponding to the generic search direction u=sinθ. Assuming the target is within a specific angular region of interest, denoted as $U_{MAX}=\left[-u_{max},u_{max}\right]$, the ML estimator is given by:

(4) $${\hat{u}}_{T}=\underset{u\in U_{\mathrm{MAX}}}{\max}\left\{V\left(u\right)\right\},$$

A theoretical performance prediction for the MSE of the ML estimator has been derived in [16], and it is briefly recalled here for the reader convenience. This performance prediction is based on the hypothesis that the MSE contribution can be decomposed in the sum two main terms:

- A small error contribution, due to distortions on the likelihood function main lobe caused by thermal noise;
- A large error contribution due to the presence of the outliers, namely DoA estimates located well outside the main lobe peak on the likelihood function, around the angles, $u_{m},\;m=1,\ldots ,M$ where it has its sidelobes’. These errors are still caused by the presence of thermal noise that occasionally makes them appear higher than the mainlobe peak.

Specifically, the MSE prediction $\zeta \left(SNR,d_{1},\;d_{2},u_{T}\right)$ is decomposed in [16] as:

(5) $$\zeta \left(SNR,d_{1},\;d_{2},u_{T}\right)≜E\left[{\left({\hat{u}}_{T}-u_{T}\right)}^{2}\right]\approx \left[1-\sum_{m=1}^{M}P_{m}\right]\cdot CRB+\sum_{m=1}^{M}{\left(u_{m}-u_{T}\right)}^{2}P_{m},$$

where

- M represents the number of sidelobe peaks in the angular sector of interest $U_{MAX}$;
- ${P_{m}=P}_{m}\left(SNR,d_{1},\;d_{2},u_{T}\right)$ represents the mth pairwise error probability, i.e., the probability that the mth sidelobe peak is higher than the main lobe peak;
- $u_{m}$ is the m-th sidelobe peak location;
- CRB is the Cramer–Rao lower bound, representing the MSE achieved when the contribution due to outliers is negligible.

For this approximation to be tight, the union bound limit must apply [25,30], meaning that the probability of two side peaks simultaneously being higher than the main lobe peak must be negligible. Clearly, this depends on both the input SNR and the likelihood function’s side peaks amplitudes, which in turn depend on the array geometry. In practice, for a given array geometry, there is a specific SNR value above which the MSE approximation is tight.

**Figure 3:**
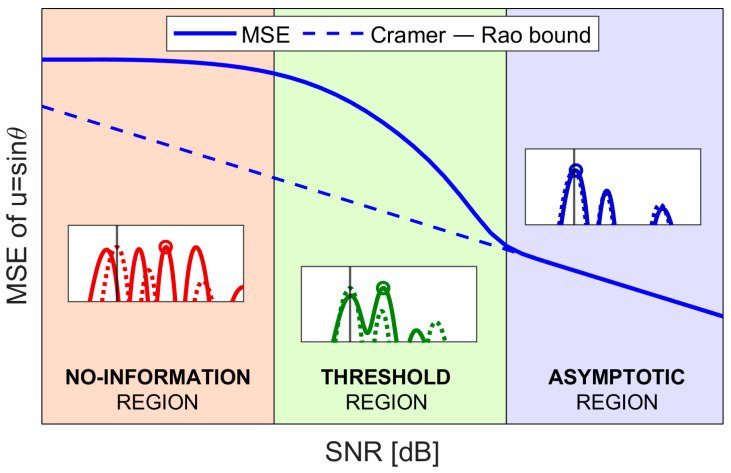
Qualitative behavior of the mean square error (MSE) vs. signal-to-noise power ratio (SNR).

This SNR value also determines the lower limit of the so-called threshold region [16], where the probability of outliers is non-negligible, and the achieved MSE deviates from the CRB. When the input SNR is high enough, the probability of outliers becomes negligible, making the CRB a good approximation of the MSE. Since in this case the asymptotic performance is reached, this SNR region is referred to as the asymptotic region in [16]. Lastly, when the SNR is too low, the union bound approximation is no longer valid, meaning that there is a non-negligible probability of multiple side peaks being higher than the main lobe peak. Under this condition, the DoA can no longer be estimated, so this SNR region is referred to as the no-information region in [16]. A qualitative overview of the three regions described is shown in Figure 3.

The goal of this paper is to control the probability of outliers in the estimates obtained by the MIMO radar by optimizing the design of the three-element array. Specifically, we aim to jointly optimize the positions of both the transmitting/receiving elements and the receiving-only elements, enabling the design of MIMO NULA configurations, which are robust to outliers, not only in the asymptotic region, but also for SNR values within the threshold region. To this end, in the next section we constrain the maximum value for the MIMO pairwise error probabilities $P_{m}$ in Equation (5), aiming to reduce the contribution of outlier estimates to the global MSE.

## 3. Admissible Pairwise Probability Region

In this section, we carry out the analytical derivations required to design outlier-robust NULA configurations, tailored for MIMO radar systems. From Equation (5), we note that the m-th pairwise error probability $P_{m}$ acts as a scaling factor for the large error contributions ${\left(u_{m}-u_{T}\right)}^{2}$ that cause the MSE to deviate from the CRB. Therefore, in the following we will call outlier-robust array configurations all those NULAs which satisfy a desired constraint on the maximum pairwise error probabilities $P_{m}$. Based on [16], the mathematical expression for the m-th pairwise error probability is:

(6) $$P_{m}=Q\left(a,b\right)-\frac{1}{2}e^{-\frac{SNR}{2}}I_{0}\left(\frac{SNR}{2}\frac{\sqrt{L\left(u\right)}}{MN}\right),$$

where

- $I_{0}$ denotes the modified Bessel function of the first kind and order zero;
- SNR is the signal-to-noise power ratio, directly related to the complex baseband a target amplitude, i.e., $SNR=A^{2}$;
- $Q\left(a,b\right)$ denotes the Marcum Q function of the two arguments

(7) $$a≜\sqrt{\frac{SNR}{2}M\left(1-\sqrt{1-\frac{L\left(u\right)}{M^{2}N^{2}}}\right)},\;b≜\sqrt{\frac{SNR}{2}M\left(1+\sqrt{1-\frac{L\left(u\right)}{M^{2}N^{2}}}\right),}$$

and

(8) $$L\left(u\right)={\left|s^{H}\left(u\right)s\left(u_{T}\right)\right|}^{2}$$

is the normalized noiseless likelihood function that depends only on the array geometry and achieves the maximum value of MN when $u=u_{T}$.

The first step of the design strategy is to impose a constraint on the maximum value of $P_{m}$, namely:

(9) $$P_{m}\leq P_{max},\;\forall m=1,\ldots ,M.$$

From Equation (6), we note that $P_{m}$ only depends on the array geometry, by means of the noiseless likelihood function, and has a monotonic behavior with the operational SNR, which determines how much this noiseless likelihood function gets distorted. Therefore, for a fixed SNR value, the constraint on the maximum admissible $P_{m}$ value can be converted into a constraint on the maximum admissible value for the m-th sidelobe peak of the normalized likelihood, namely $L\left(u_{m}\right)\leq L_{max}$, where the maximum admissible value $L_{max}$ can be univocally determined by inverting Equation (6) for a given SNR value.

The problem then becomes to identify all the array configurations with M transmit elements and N receive elements that satisfy the constraint $L\left(u_{m}\right)\leq L_{max}$. To this end, we define the angles

(10) $$\phi_{k}≜2\pi \frac{d_{k}}{\lambda }\left(u-u_{T}\right),$$

and express $L\left(u\right)$ for the generic N and M as:

(11) $$L\left(u\right)={\left|\sum_{m=0}^{M-1}\sum_{n=0}^{N-1}e^{j2\pi \frac{d_{m}^{TX}+d_{n}}{\lambda }\left(u-u_{T}\right)}\right|}^{2}=\Psi_{M}\left(\phi_{0}^{TX},\cdots ,\phi_{M-1}^{TX}\right)\cdot \Psi_{N}\left(\phi_{0},\cdots ,\phi_{N-1}\right),$$

where for the generic number of elements K:

(12) $$\Psi_{K}\left(\phi_{0},\cdots ,\phi_{K-1}\right)={\left|\sum_{k=0}^{K-1}e^{j\phi_{k}}\right|}^{2}$$

In the following, taking the phase reference in the most left-side element n=0, it is assumed for simplicity $\phi_{0}=0$, so that $\phi_{0}$ is not listed among the variables of the functions $\Psi_{N}$ and $\Psi_{M}$.

As is apparent, we successfully decoupled the contributions of the phase shifts due to the transmitter-to-target path, encapsulated in $\Psi_{M}\left(\phi_{0}^{TX},\cdots ,\phi_{M-1}^{TX}\right)$, from those due to the target-to-receiver path, encapsulated in $\Psi_{N}\left(\phi_{0},\cdots ,\phi_{N-1}\right)$. Based on Equation (11), we can derive the outlier-robust NULA design strategy for MIMO radar configurations. In the following subsections, three different setups are considered:

- A 3 × 3 MIMO setup with three trans-receiving array elements;
- A 2 × 3 MIMO setup with two trans-receiving array elements, and a third receiving-only element;
- A SIMO setup with a single trans-receiving element and two receiving-only elements.

### 3.1. 3 × 3 MIMO with Three Trans-Receiving Array Elements

Based on Equation (11), in the 3 × 3 MIMO setup, N=M=3, $d_{m}^{TX}=d_{m},\;m=0,\cdots ,M-1$, and the transmission functional is identical to the reception functional, so that the constraint $L\left(u\right)\leq L_{max}$ becomes:

(13) $$L\left(\phi_{1},\phi_{2}\right)={\left|1+e^{j\phi_{1}}+e^{j\phi_{2}}\right|}^{4}$$

We notice that this functional has a large number of symmetries among its angles, among which:

(14) $$L\left(\phi_{1},\phi_{2}\right)=L\left(\phi_{2},\phi_{1}\right)=L\left(\phi_{2}-\phi_{1},\phi_{2}\right)=L\left(\phi_{1}-\phi_{2},{-\phi }_{2}\right)=L\left(-\phi_{1},\phi_{2}-\phi_{1}\right)=L\left(\phi_{1},\phi_{1}-\phi_{2}\right)$$

By defining a 45° rotated axis system as:

(15) $$\left\{\begin{array}{c} x=\frac{\phi_{2}-\phi_{1}}{2}\\ y=\frac{\phi_{1}+\phi_{2}}{2} \end{array}\right.,\;\left\{\begin{array}{c} \phi_{1}=y-x\\ \phi_{2}=y+x \end{array}\right.$$

and following the derivations in Appendix A, we obtain the following mathematical representation of the set of points identified by cutting the normalized likelihood function at the value $L_{max}$ in the $\left(\phi_{1},\phi_{2}\right)$ plane.

(16) $$\left\{\begin{array}{c} \phi_{1}=\pm \mathrm{acos}\left[\frac{\sqrt{L_{max}}-1}{4\cos x}-\cos x\right]+2\pi h-x\\ \phi_{2}=\pm \mathrm{acos}\left[\frac{\sqrt{L_{max}}-1}{4\cos x}-\cos x\right]+2\pi k+x \end{array}\right.,$$

with the constraint

(17) $$-\mathrm{acos}\left(\frac{-1+\sqrt[{4}]{L_{max}}}{2}\right)<x\leq \mathrm{acos}\left(\frac{-1+\sqrt[{4}]{L_{max}}}{2}\right).$$

**Figure 4:**
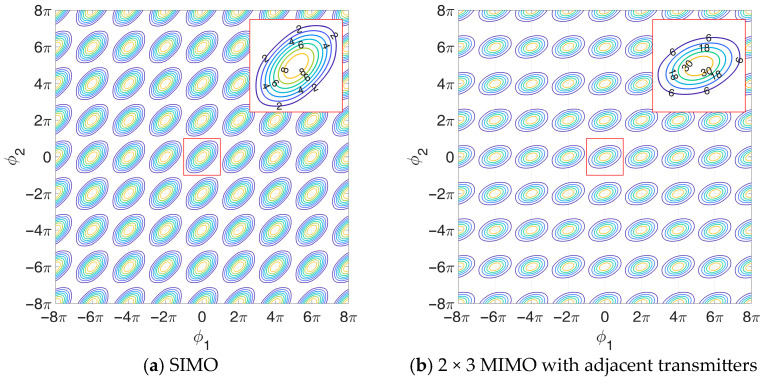

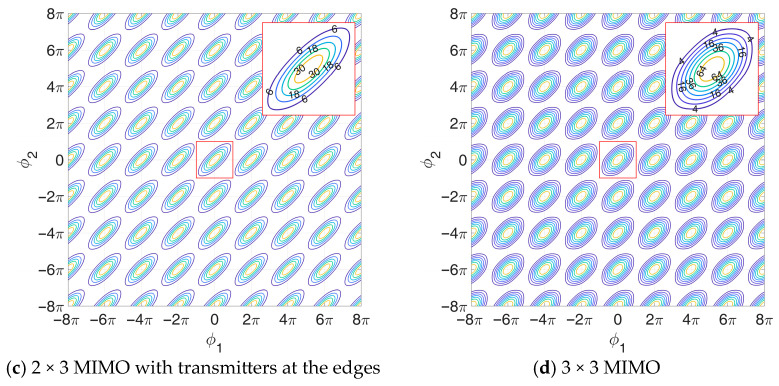
Contour plot of the noiseless normalized likelihood function $L(\phi_{1},\phi_{2})$: (**a**) SIMO; (**b**) 2 × 3 MIMO with adjacent transmitters; (**c**) 2 × 3 MIMO with external transmitters; (**d**) 3 × 3 MIMO.

A contour plot of the likelihood function for the 3 × 3 MIMO case is shown in Figure 4d.

### 3.2. 2 × 3 MIMO with Two Trans-Receiving Array Elements

The 2 × 3 MIMO case can be divided into three subcases, where the single receiving-only element is positioned alternately at the leftmost, rightmost, or central position. To mathematically represent the constraint $L\left(u\right)\leq L_{max}$ in the $\left(\phi_{1},\phi_{2}\right)$ plane when only two of the array elements are equipped with a transmitter, we conveniently set the phase reference at the receiving-only element, as this simplifies the derivation. The three subcases of the 2 × 3 MIMO configuration can be readily obtained from one another through a simple coordinate transformation in the $\left(\phi_{1},\phi_{2}\right)$ plane.

By setting M=2, $d_{0}^{TX}=d_{1},\;d_{1}^{TX}=d_{2},$ and taking the phase reference at the receiving-only element the transmission functional for the 2 × 3 MIMO case is given by:

(18) $$\Psi_{2}\left(\phi_{0}^{TX},\phi_{1}^{TX}\right)=\Psi_{2}\left(\phi_{1},\phi_{2}\right)={\left|e^{j\phi_{1}}+e^{j\phi_{2}}\right|}^{2},$$

while the reception functional is the same as in the 3 × 3 MIMO case. Therefore, we obtain:

(19) $$L\left(u\right)=\Psi_{2}\left(\phi_{0}^{TX},\phi_{1}^{TX}\right)\cdot \Psi_{3}\left(\phi_{1},\phi_{2}\right)={\left|e^{j\phi_{1}}+e^{j\phi_{2}}\right|}^{2}{\left|1+e^{j\phi_{1}}+e^{j\phi_{2}}\right|}^{2}\leq L_{max}.$$

The derivation details are provided in Appendix B, while here we only report the expressions to mathematically characterize the set of points in $\left(\phi_{1},\phi_{2}\right)$, identified by cutting the normalized likelihood function at $L_{max}$:

(20) $$\left\{\begin{array}{c} \phi_{1}=\pm \mathrm{acos}\left[\frac{\frac{L_{max}}{4\cos^{2}x}-1}{4\cos x}-\cos x\right]+2\pi h-x\\ \phi_{2}=\pm \mathrm{acos}\left[\frac{\frac{L_{max}}{4\cos^{2}x}-1}{4\cos x}-\cos x\right]+2\pi k+x \end{array}\right.,$$

with

(21) $$-\mathrm{acos}\left(-\frac{1-\sqrt{1+4\sqrt{L_{max}}}}{4}\right)<x\leq \mathrm{acos}\left(-\frac{1-\sqrt{1+4\sqrt{L_{max}}}}{4}\right),$$

Finally, we describe how to transition between different 2 × 3 MIMO configurations.

For the 2 × 3 MIMO case with the receiving-only antenna at the rightmost array element, using M=2, $d_{0}^{TX}=d_{0},\;d_{1}^{TX}=d_{1}$ in Equation (12) yields:

(22) $$\Psi_{2}\left(\phi_{0}^{TX},\phi_{1}^{TX}\right)=\Psi_{2}\left(\phi_{1}\right)={\left|{1+e}^{j\phi_{1}}\right|}^{2},$$

while the reception functional is the same as in the square root of the 3 × 3 MIMO case. Therefore, we obtain:

(23) $$L\left(u\right)=\Psi_{2}\left(\phi_{0}^{TX},\phi_{1}^{TX}\right)\cdot \Psi_{3}\left(\phi_{1},\phi_{2}\right)={\left|1+e^{j\phi_{1}}\right|}^{2}{\left|1+e^{j\phi_{1}}+e^{j\phi_{2}}\right|}^{2}\leq L_{max}.$$

It is easy to notice that the configuration is perfectly symmetric to the previous one. Therefore, the resulting coordinate transformation is straightforward. Specifically, by defining $\phi_{1}^{\prime }≜\phi_{2}-\phi_{1},\;\phi_{2}^{\prime }≜\phi_{2}$, we can transform the TX functional in the TX functional of the previous case, while the RX functional remains unchanged thanks to its symmetry properties listed above.

Based on this coordinate transformation, we can reuse Equations (20) and (21) to describe the set of points in the $\left(\phi_{1},\phi_{2}\right)$ plane that correspond to the normalized likelihood function being cut at $L_{max}$ in the 2 × 3 MIMO case with a receiving-only antenna at the rightmost element.

Similarly, for the 2 × 3 MIMO case where the external elements of the array are used as transmitters and the receiving-only element is at the center, using M=2, $d_{0}^{TX}=d_{0},\;d_{1}^{TX}=d_{2}$ in Equation (12) yields:

(24) $$\Psi_{2}\left(\phi_{0}^{TX},\phi_{1}^{TX}\right)=\Psi_{2}\left(\phi_{1}\right)={\left|{1+e}^{j\phi_{1}}\right|}^{2},$$

while the reception functional is the same as in the square root of the 3 × 3 MIMO case. Therefore, we obtain:

(25) $$L\left(u\right)=\Psi_{2}\left(\phi_{0}^{TX},\phi_{1}^{TX}\right)\cdot \Psi_{3}\left(\phi_{1},\phi_{2}\right)={\left|1+e^{j\phi_{2}}\right|}^{2}{\left|1+e^{j\phi_{1}}+e^{j\phi_{2}}\right|}^{2}\leq L_{max}.$$

Although this case has no direct relationship with the previous ones, we notice that it can be obtained from the first one by defining $\phi_{1}^{\prime \prime }≜\phi_{2}-\phi_{1},\;\phi_{2}^{\prime \prime }≜-\phi_{1}$, which again transforms Equation (24) in the TX functional of the first case, while the RX functional remains unchanged thanks to its symmetry properties listed above. Contour plots of the likelihood function in the 2 × 3 MIMO cases are shown in Figure 4b,c. Specifically, in Figure 4b, we assume adjacent transmitters, with the receiving-only antenna located at the leftmost array element. The symmetric case is not included, as the likelihood function appears identical. Furthermore, Figure 4d shows the likelihood function contour plot obtained in the 2 × 3 MIMO case with transmitters located at the edges of the array.

### 3.3. SIMO with a Single Trans-Receiving Array Elements

Finally, we briefly describe how the mathematical formulation of the constraint $L\left(u\right)<L_{max}$ changes in the SIMO case. A detailed derivation along these lines is provided in our previous work [29]. Specifically, when only a single transmitter is present (M=1), and if the transmitter corresponds to the leftmost array element, M = 1, $d_{0}^{TX}=d_{0}$, the transmission functional $\Psi_{M}\left(\phi_{m}\right)$ simplifies to:

(26) $$\Psi_{1}\left(\phi_{0}\right)={\left|e^{j\phi_{0}}\right|}^{2}=\Psi_{1}=1.$$

Therefore, in the SIMO case the normalized likelihood function only depends on the reception functional $\Psi_{N}\left(\phi_{n}\right)$:

(27) $$L\left(u\right)=\Psi_{N}\left(\phi_{n}\right)={\left|1+e^{j\phi_{1}}+e^{j\phi_{2}}\right|}^{2}\leq L_{max}.$$

Here, the normalized likelihood function is equal to the square root of the one in Equation (13), obtained in the 3 × 3 MIMO case. Therefore, the mathematical representation of the set of points identified by cutting the normalized likelihood function at $L_{max}$ is obtained from that of the SIMO case by substituting $\sqrt{L_{max}}$ with $L_{max}$:

(28) $$\left\{\begin{array}{c} \phi_{1}=\pm \mathrm{acos}\left[\frac{L_{max}-1}{4\cos x}-\cos x\right]+2\pi h-x\\ \phi_{2}=\pm \mathrm{acos}\left[\frac{L_{max}-1}{4\cos x}-\cos x\right]+2\pi k+x \end{array}\right.,$$

with

(29) $$-\mathrm{acos}\left(\frac{-1+\sqrt{L_{max}}}{2}\right)<x\leq \mathrm{acos}\left(\frac{-1+\sqrt{L_{max}}}{2}\right).$$

Figure 4d shows the likelihood function contour plot obtained in the SIMO case.

By comparing the contour plots in Figure 4, we note that the different likelihood functions obtained for the considered MIMO cases appear distorted compared to the one for SIMO. This is since the dependency of the $\Psi_{M}\left(\phi_{1},\phi_{2}\right)$ functional on $\phi_{1}$ and $\phi_{2}$ changes depending on the transmitter positioning. Notably, the 3 × 3 MIMO (Figure 4d) has the same shape as the one for the SIMO (Figure 4a), since in this case the transmission and reception functionals are the equal, so that the 3 × 3 MIMO likelihood function is simply the square of the SIMO likelihood function.

### 3.4. From the Likelihood Function Contours to the Admissible Region

After describing the likelihood functions for all the MIMO design case studies, we need to convert the derived mathematical representations into suitable admissible regions in the inter-element distance plane. This can be done by following a simple procedure, similar to the one in [29]. Particularly, Equation (10) allows us to map the quantities $\phi_{1}$ and $\phi_{2}$ into inter-element distances. This requires knowing the maximum range of variation of $\left(u-u_{T}\right)$, which depends on the extremes of the surveilled angular sector $U_{max}$. Specifically, we have:

(30) $$-u_{max}\leq u-u_{T}\leq u_{max}.$$

The value of $u_{max}$ acts as a scale factor for the axes, allowing us to rewrite $\phi_{1}$ and $\phi_{2}$ in terms of the two inter-element distances:

(31) $$\frac{d_{k}}{\lambda }=\frac{\phi_{k}}{2\pi }\cdot \frac{1}{u_{max}}.$$

After this scaling, the mathematical representation of the set of points identified by the cut of the normalized likelihood function at the value $L_{max}$ can be used to obtain an admissible pairwise probability (APP) region in the plane of inter-element distances including all the array configurations satisfying the constraint $L\left(u_{m}\right)<L_{max}$.

**Figure 5:**
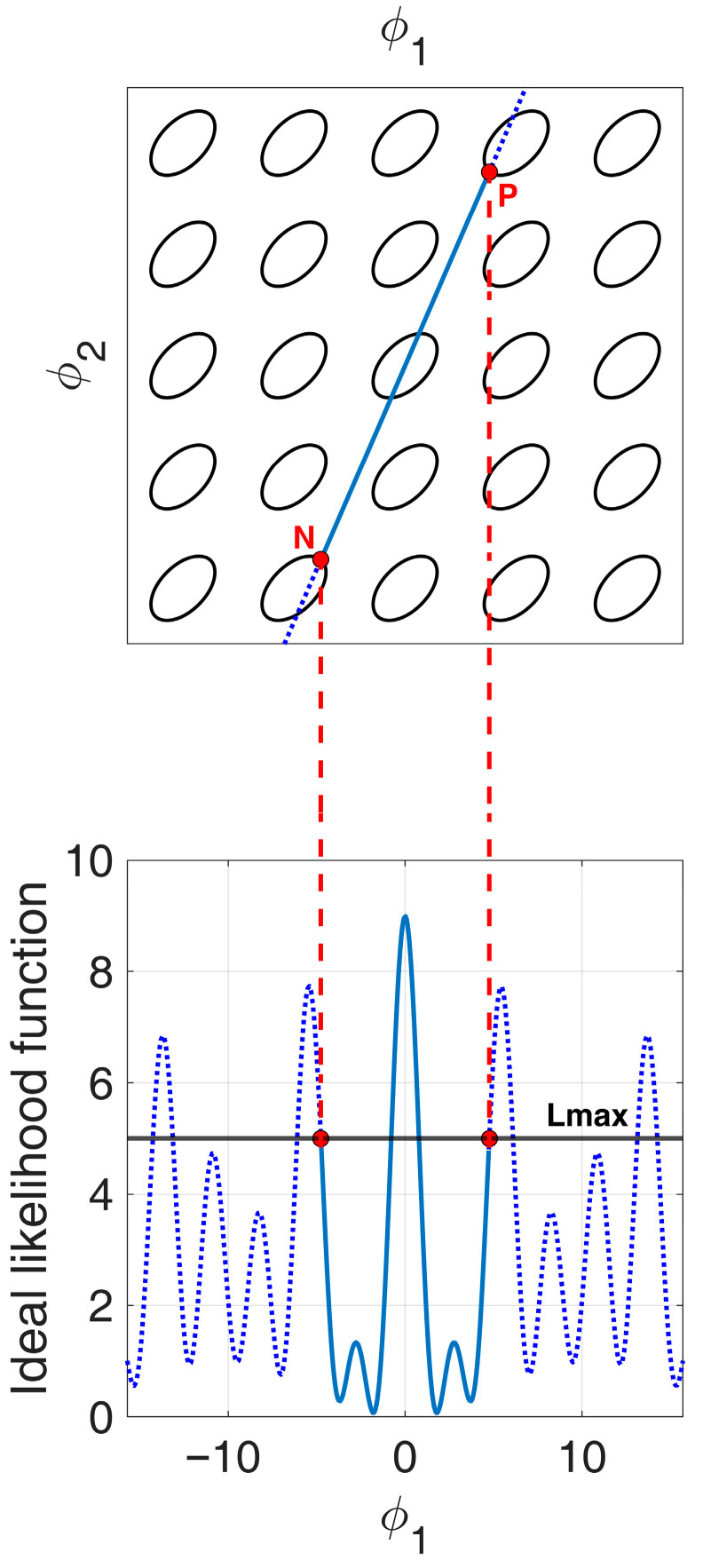
Likelihood function $L\left(\phi_{1},\phi_{2}\right)$ cut at $L_{max}$. The linear segment $\bar{NP}$ with slope α represents the beampattern of a specific 3-element array $\bar{z}=\left[0,{\bar{d}}_{1},\alpha {\bar{d}}_{1}\right]$.

The example in Figure 5 allows us to better clarify the procedure to obtain the APP region.

We note that the locus of points corresponding to the array beampattern of a specific 3-element array $\bar{z}=\left[0,{\bar{d}}_{1},\alpha {\bar{d}}_{1}\right]$ can be represented on the $\left(\phi_{1},\phi_{2}\right)$ plane as a linear segment $\bar{NP}$, as shown in Figure 5. The length of the segment is related to the total length of the array $\bar{z}$, while its slope α is determined based on the relationship between the two inter-element distances ${\bar{d}}_{1}$ and ${\bar{d}}_{2}$. Specifically, based on Equation (10), we have:

(32) $$\alpha ≜\frac{{\bar{\phi }}_{2}}{{\bar{\phi }}_{1}}=\frac{{\bar{d}}_{2}}{{\bar{d}}_{1}},$$

so that, for instance, all ULA configurations correspond to segments that lie on the line with slope α=2, since for every three-element ULA we have $d_{2}=2d_{1}$.

For a fixed value $L_{max}$, for increasing values of $\phi_{1}$, the first intersection with $L\left(\phi_{1},\phi_{2}\right)$ along the slope α represents a specific solution $\left({\bar{\phi }}_{1},{\bar{\phi }}_{2}\right)$ of the equation

(33) $$L\left(\phi_{1},\phi_{2}\right)=L_{max}.$$

For values of $\phi_{1}$ greater than ${\bar{\phi }}_{1}$ along the slope α, the constraint $L\left(\phi_{1},\phi_{2}\right)\leq L_{max}$ is violated. This is visible in Figure 5, where the beampattern of the array $\bar{z}$ is shown as a function of $\phi_{1}$.

Based on the considerations made in Figure 5, the APP region can be obtained by following the procedure outlined in [29], and recalled here:

- Inside the set of all possible slopes $A=\left[1,+\infty \right]$, determine the subset of slopes α blocked by the first-order replica of $L\left(\phi_{1},\;\phi_{2}\right)$, i.e., $\left(k,h\right)=\left(0,1\right)$. Notice that all the replicas $\left(k,h\right)=\;(0,h)$ are blocked by replica (0,1), so they will be ignored in the following.
- Update the set of the non-blocked slopes A after step I.
- Inside the set A, determine the subset of slopes α blocked by the next higher-order replica of $L\left(\phi_{1},\;\phi_{2}\right)$, i.e., $\left(k,h\right)=\;(1,1)$. Notice that the replicas $\left(k,h\right)=\;(h,h)$ are blocked by replica (1,1), so they will be ignored in the following.
- Update the set of the non-blocked slopes A after step III.
- Inside the set A, determine the subset of slopes α blocked by higher-order replicas of $L\left(\phi_{1},\;\phi_{2}\right)$.
- Update the set of the non-blocked slopes A after step V.
- Repeat steps V and VI until the set A is empty.

The presented procedure could be extended to N=4 antenna elements, by operating in a 3D space, where the contours are replaced by surfaces. While the extension is feasible, the analytical derivation would make the description cumbersome. Therefore, we avoid an explicit illustration of this case and leave it to the interested reader.

**Figure 6:**
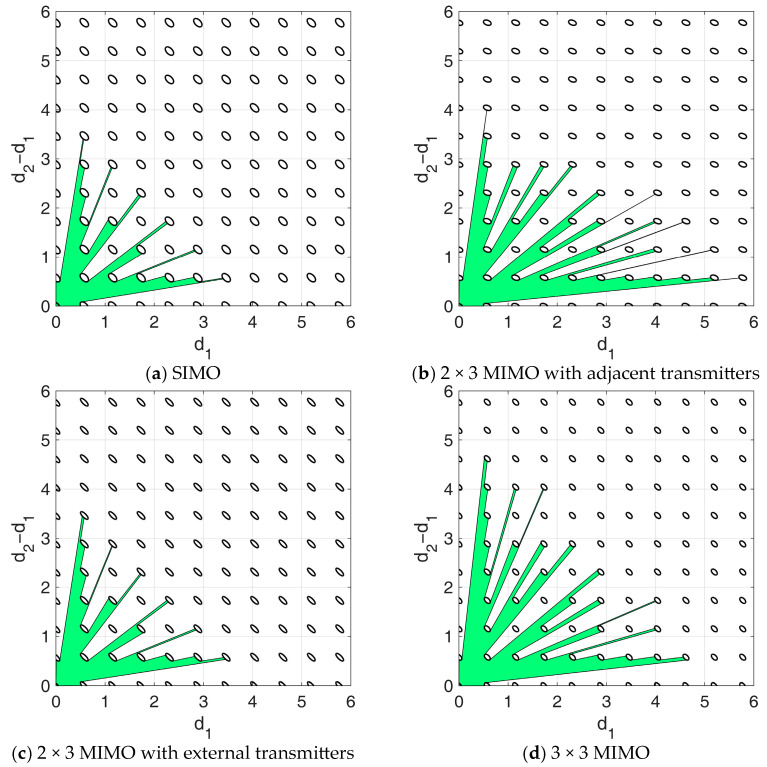
Admissible regions obtained assuming SNR=15 dB, $P_{max}=1\cdot 10^{-3}$, $U_{max}=\left[-60{}^{\circ},60{}^{\circ}\right]$. (**a**) SIMO; (**b**) 2 × 3 MIMO with adjacent transmitting elements (the transpose applies when using elements 1 and 2 as TX instead of elements 0 and 1); (**c**) 2 × 3 MIMO with external transmitting elements; (**d**) 3 × 3 MIMO.

Using this procedure, the likelihood function contours in Figure 4 can be easily converted into equivalent APP regions, containing all the array configurations that satisfy the constraint $P_{m}\leq P_{max}$. Figure 6a–d show the admissible regions obtained from the likelihoods in Figure 4, in the 2D domain $\left(d_{1},d_{2}-d_{1}\right)$ of the two inter-element distances, assuming SNR=15 dB, $P_{max}=1\;\cdot 10^{-3}$, and $U_{max}=\left[-60{}^{\circ},60{}^{\circ}\right]$, along with the likelihood function cut at $L_{max}$. As expected, the APP region appears symmetrical in this domain when using the 3 × 3 MIMO configuration, the 3 × 1 SIMO configuration and the 3 × 2 MIMO with the external transmitting elements. This is clearly not the case for the 3 × 2 MIMO configurations with adjacent transmitting elements, because the transmitters insisting only on one of the two baselines makes the behavior unsymmetric.

In conclusion, in this section, we derived the mathematical expression to identify an APP region in the plane of the two inter-element distances for each MIMO case considered. The derived APP region contains all the NULA configurations that satisfy the constraint imposed on the pairwise error probabilities, allowing us to design outlier-robust three-element NULA configurations based on the design parameters, namely the operational SNR, the maximum pairwise error probability, and the angular sector of interest. An example of application of the proposed strategy is shown in the next section, where we compare the DoA estimation accuracy and the robustness to outliers of different NULA configurations resulting from the proposed design strategy.

## 4. A Design Case Study of the Proposed Strategy

In this section, we show an application example of the design strategy introduced in the previous section, and we compare different NULA designs considering both SIMO and MIMO operation. Particularly, we assume the same design parameters used in the previous section, namely SNR=15 dB, $P_{max}=1\cdot 10^{-3}$, and $U_{max}=\left[-60{}^{\circ},60{}^{\circ}\right]$. We further assume transmitting narrowband orthogonal signals in the Wi-Fi bandwidth, with a carrier frequency of $f_{0}=2.4\;GHz$, corresponding to a wavelength of λ≈0.125 m.

Before proceeding with the design, we note that the results in this section also relax the assumption of perfect waveform orthogonality. Specifically, while the theoretical MSE curves are obtained using the theoretical prediction derived in [16] and reported in Equation (5), they are compared with Monte Carlo simulation results. For these simulations, we considered a set of quasi-orthogonal LFM waveforms (up-chirp, down-chirp, and V-chirp), each with a time-bandwidth product of 200 (B=20 MHz, T=10µs). This allows us to demonstrate that the non-perfect orthogonality between waveforms does not significantly alter the validity of our theoretical analysis. Specifically, due to the high compression gain, the energy of the residual cross-talk after range compression is negligible compared to the main auto-correlation peak.

**Figure 7:**
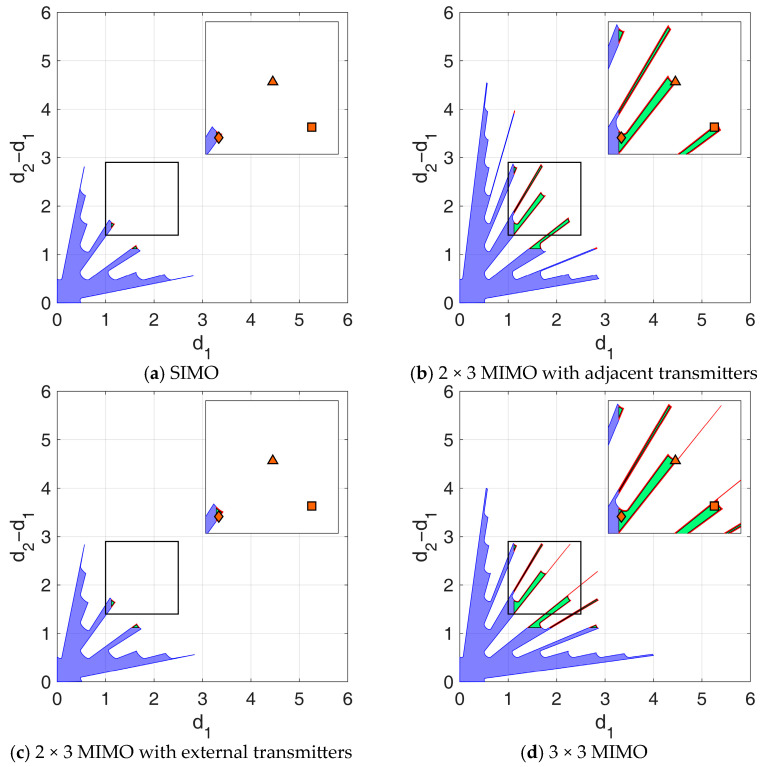
Admissible regions obtained assuming SNR=15 dB, $P_{max}=1\cdot 10^{-3}$, $U_{max}=\left[-60{}^{\circ},60{}^{\circ}\right]$, and considering additional technological constraints (antenna size: 14 cm, mounting tolerance: 1 cm). (**a**) SIMO; (**b**) 2 × 3 MIMO with adjacent transmitters; (**c**) 2 × 3 MIMO with transmitters at the edges; (**d**) 3 × 3 MIMO.

Similar to [29], the proposed design strategy is particularly flexible, as the identified APP region eventually allows the designer to account for additional technological constraints that may emerge during the design phase. Particularly, in this section, the APP regions resulting from the design procedure are further adjusted to account for two additional technological constraints:

- Minimum inter-element distance: To account for the physical size of the antennas (assumed to be 14 cm), we enforce a minimum inter-element distance of l≈1.12λ. This constraint, which excludes the blue area in Figure 7 from the APP region, carries the important secondary benefit of mitigating mutual coupling, since the electromagnetic interaction between elements is significantly reduced at large element separations.
- Mounting tolerance: Since the antennas cannot be positioned with infinite precision, we assume a mounting tolerance of 1.5 mm in each direction, corresponding to δ=0.012λ in wavelength units. This requires excluding from the APP region the areas close to the region border (i.e., the red region in Figure 7).

These two constraints modify the admissible regions in Figure 6 as shown in Figure 7. The following observations are in order:

- As shown in Figure 6a, the SIMO configuration already achieved the smallest APP region compared to MIMO setups (in Figure 6b–d). Therefore, the additional technological constraints in this case severely limit the set of admissible NULA configurations, as the residual APP region (Figure 7a) becomes extremely small, leaving very little to no flexibility in the array design choice.
- By bringing the available two-way phase centers closer together, the MIMO 2 × 3 with adjacent transmitters prioritizes outlier robustness. This results in a larger APP region even after technological constraints (Figure 7b), providing significant flexibility and greater design freedom compared to SIMO.
- In contrast, the MIMO 2 × 3 with transmitters at the edges of the array (Figure 7c) increases the overall separation between the array two-way phase centers, resulting in a narrower main lobe in the likelihood function. This leads to improved performance in the high SNR conditions, prioritizing asymptotic estimation accuracy over outlier robustness. Therefore, the resulting APP region is very similar to the one obtained in the SIMO case.
- Finally, the MIMO 3 × 3 (Figure 7d) benefits from the use of three transmitters, providing more balanced performance. Since the available power now is equally split among nine different two-way phase centers, we can benefit from both improved asymptotic estimation accuracy and enhanced robustness to outliers, resulting in the largest admissible region.

Based on the above observations, we conduct two different types of comparisons between the different SIMO and MIMO configurations:

1. Comparison with a fixed array configuration: We analyze the performance of the NULA $z_{1}=\left[0\;1.16\;2.77\right]\lambda$ (‘◊’ marker), which is admissible in all SIMO/MIMO APP regions.
2. Comparison with a fixed maximum pairwise error probability: We compare the performance of $z_{1}=\left[0\;1.16\;2.77\right]\lambda$ (‘◊’ marker) for the SIMO and the 2 × 3 MIMO configuration with transmitters at the edges, $z_{2}=\left[0\;2.20\;3.91\right]\lambda$ (‘□’ marker) for the 2 × 3 MIMO with adjacent transmitters, and $z_{3}=\left[0\;1.76\;3.98\right]\lambda$ (‘∆’ marker) for the 3 × 3 MIMO. All configurations ensure a maximum pairwise error probability of approximately $P_{max}\approx 0.6\cdot 10^{-3}$.

**Figure 8:**
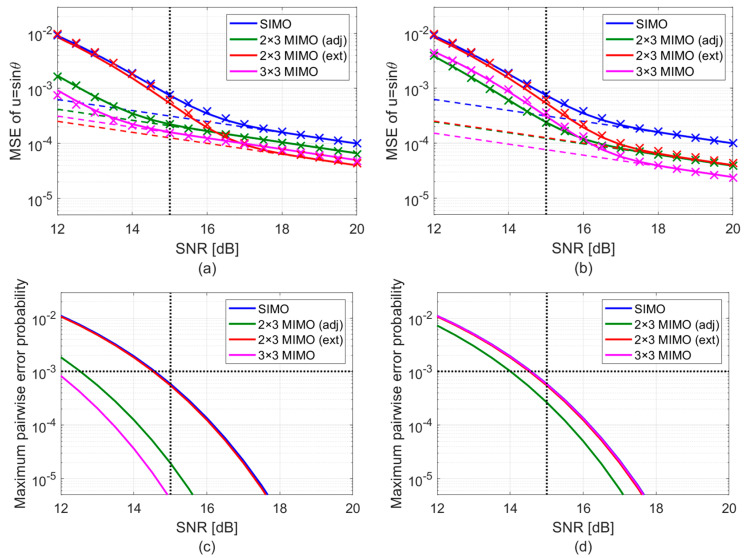
DoA estimation MSE and maximum pairwise error probability as a function of the SNR. The comparisons are performed (**a**,**c**) assuming the same array configuration $z_{1}$, achieving different maximum pairwise error probabilities for different SIMO/MIMO configurations, and (**b**,**d**) assuming different array configurations achieving the same maximum pairwise error probability, $P_{max}\approx 0.6\cdot 10^{-3}$.

The first comparison highlights two key advantages of MIMO: (i) the ability to control outlier probability and (ii) the improved asymptotic performance of MIMO configurations compared to SIMO, which results from an increase in the total effective array length. Specifically, Figure 8a shows the MSE of the different setups, averaged within the $U_{max}$ sector, as a function of SNR, while Figure 8c shows the probability of outlier and the maximum pairwise error probability as a function of the SNR. The following observations are in order:

- When the SNR is within the threshold region, the MSE obtained with array $z_{1}$ progressively improves from SIMO to MIMO. Specifically, the 3 × 3 MIMO achieves the lowest MSE. Instead, the MSE of the 2 × 3 MIMO configuration with external transmitters is relatively close to the SIMO, since the probability of an outlier is still significant at this SNR value. This is also clearly visible from the red curve Figure 8c.
- As the SNR increases and the performance gets closer to the asymptotic region, the impact of outliers on the MSE becomes negligible (see Figure 8d). As a result, the asymptotic term in Equation (6) becomes the dominating MSE contribution. Therefore, for high SNR values, e.g., at SNR=20 dB where all four array configurations achieve asymptotic MSE performance, the 2 × 3 MIMO configuration with external transmitters outperforms all other setups, since it yields the lowest CRB. Conversely, as the CRB of the 2 × 3 MIMO with adjacent transmitters is only marginally better than that of SIMO, for high SNR values the green MSE curve representing the 2 × 3 MIMO setup with adjacent transmitters gets closer to the SIMO performance. Finally, the 3 × 3 MIMO configuration provides a more balanced performance, as it distributes the available transmit power across nine different phase centers.
- As discussed at the beginning of this section, the markers (‘×’) in Figure 8 denote Monte Carlo simulation results obtained using quasi-orthogonal LFM waveforms. Given the high compression gain of these waveforms, the slight residual cross-talk is negligible. As a result, the simulated points show excellent consistency with the theoretical predictions, which assume perfect orthogonality. This further confirms the effectiveness of the proposed model and approach.

Overall, the proposed array design ensures high estimation accuracy for SNR values equal to or greater than the design SNR, maintaining an estimation accuracy close to the CRB.

To complement this analysis, we conduct an additional comparison at a fixed-pairwise error probability. Specifically, the potential of the 2 × 3 MIMO with adjacent transmitters and the 3 × 3 MIMO setups is not fully exploited. Although we ensured a maximum pairwise error probability of $10^{-3}$ at a design SNR of 15 dB, using array $z_{1}$, in these two cases, results in a much lower actual pairwise error probability, namely $5.4\cdot 10^{-6}$ for the 2 × 3 MIMO with adjacent transmitter, and $5.2\cdot 10^{-6}$ for the 3 × 3 MIMO. This explains the superior performance of these two configurations at the design SNR. However, the constraint was overfulfilled, meaning that with $z_{1}$, these setups are already very close to their CRB. Based on this analysis, we conduct a second comparison, assuming a fixed outlier probability, thus selecting a different array from the APP region in each case to ensure the same maximum pairwise error probability. Specifically:

- For SIMO and 2 × 3 MIMO with transmitters at the edges, we keep the array configuration $z_{1}$ (one of the few arrays in the APP region for these cases), which achieves a maximum pairwise error probability of about $0.6\cdot 10^{-3}$.
- For 2 × 3 MIMO with adjacent transmitters, we use array $z_{2}=\left[0\;2.20\;3.91\right]\lambda$.
- For 3 × 3 MIMO, we use array $z_{3}=\left[0\;1.76\;3.98\right]\lambda$.

Both $z_{2}$ and $z_{3}$ still achieve $P_{max}\approx 0.6\cdot 10^{-3}$, bringing them much closer to the constraint $P_{max}\leq 10^{-3}$.

Figure 8b shows the MSE of the different array configurations, averaged within the $U_{max}$ sector, as a function of SNR, while Figure 8d shows the probability of outlier and the maximum pairwise error probability as a function of the SNR. The following observations are in order:

- At the design SNR, all MSE values deviate from their respective CRBs. In such conditions, the 3 × 3 MIMO achieves the best performance due to its lower CRB, while the SIMO exhibits the highest MSE.
- Unlike in Figure 8a, in this case, the threshold region starts at approximately the same SNR for all configurations. Once asymptotic performance is reached, the 3 × 3 MIMO clearly outperforms the 2 × 3 MIMO, which in turn outperforms the SIMO. This improvement is due to the greater design flexibility provided by MIMO configurations compared to SIMO, which allows increasing the global array length, thus achieving a lower CRB.
- As observed in the fixed-array case study, the close correspondence between the theoretical predictions (assuming perfect orthogonality) and the simulation results (based on quasi-orthogonal LFM waveforms) again demonstrates the robustness of our design approach against small residual cross-talk between the transmitted waveforms.

Overall, the analysis carried out in Figure 8 highlights the twofold advantage of MIMO operation. On one hand, MIMO operation allows increasing the global array length, as it allows us to operate with an effective array with MN elements. In addition to this trivial benefit, MIMO also offers greater design flexibility, allowing us to design more challenging NULA configurations with higher global lengths. The compound effects of these two factors become evident when comparing performance under fixed-array conditions versus fixed maximum pairwise error probability, demonstrating both the structural and design benefits of MIMO in DoA estimation.

Incidentally, we observe that the sparse nature of the designed arrays may impose a lower bound on the operational range due to far-field assumptions. For instance, it is easy to verify that the longest baseline considered (i.e., D=3.98λ for array $z_{3}$) corresponds to a Fraunhofer distance of approximately 4 m at $f_{c}=2.4\;GHz$. This makes this array design best-suited for medium-to-long-range applications, where targets of interest are located at ranges from tens of meters to kilometers. For scenarios requiring operation at very short ranges, the designer could simply add a geometric constraint to the APP region to exclude arrays with excessively large apertures.

To conclude the analysis, in Table 1, we report the average MSEs achieved by different configurations in the fixed-array and fixed-pairwise error probability comparisons.

From the table, it is evident that the MSE decreases significantly when MIMO operational modes are used, both at SNR=15 dB and SNR=20 dB. To better illustrate the performance improvement of different MIMO configurations relative to SIMO, Table 2 reports the ratio between the average MSE obtained in the SIMO case and that achieved in each MIMO setup, quantifying the efficiency gains provided by MIMO in terms of estimation accuracy.

Table 2 clearly demonstrates the superior performance of MIMO configurations compared to SIMO across all considered scenarios. Particularly:

- In the fixed-array comparison at SNR = 15 dB, the 3 × 3 MIMO setup achieves the most significant reduction in MSE, with a performance improvement of 7.3 times over SIMO. The 2 × 3 MIMO with adjacent transmitters outperforms the 2 × 3 MIMO with transmitters at the edges, achieving an MSE reduction of 4.6 times compared to SIMO, thanks to its greater robustness to outliers. Finally, the 2 × 3 MIMO with transmitters at the edges offers a more modest improvement of 1.2 times, which is essentially due to its lower CRB, as the outlier probability is not different from that achieved by the SIMO.
- At SNR = 20 dB, all the setups achieve asymptotic performance, so the performance gap between configurations narrows, since the probability of outliers is negligible here. In this case, MIMO remains advantageous with respect to SIMO. Particularly, the 2 × 3 MIMO with transmitters at the edges achieves the highest improvement, showing an MSE 2.5 times lower than that achieved using SIMO.
- In the fixed-pairwise error probability comparison, the performance improvements in the threshold region, namely at SNR = 15 dB, are more similar across the different MIMO setups, since the arrays have been chosen to guarantee a similar probability of outliers across the different setups.
- At SNR = 20 dB, however, the performance improvement increases compared to the fixed-array comparison, since the MIMO setup allowed choosing longer NULA designs. Particularly, the 3 × 3 MIMO significantly outperforms the other configurations, with an MSE improvement factor of 4.2 compared to SIMO, followed by the 2 × 3 MIMO with adjacent transmitters (3.0) and the 2 × 3 MIMO with edge transmitters (2.5).

Overall, these results confirm the dual advantage of MIMO operation. First, MIMO enables an increase in the number of equivalent phase centers, leading to enhanced asymptotic performance. Second, it provides greater design flexibility, improving robustness against outliers and allowing for the implementation of NULA that would not be admissible in a SIMO setup.

**Figure 9:**
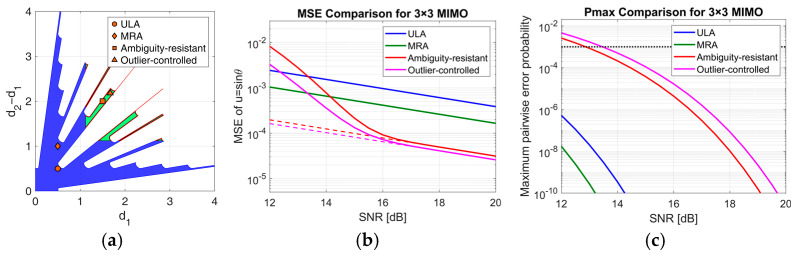
(**a**) APP region and sparse array designs relative positions in the inter-element distance plane. Comparison of (**b**) DoA estimation MSE and (**c**) maximum pairwise error probability obtained using different sparse array configurations as a function of the SNR.

After the comparison between SIMO and MIMO setups, we now compare the proposed outlier-controlled NULA design against several standard sparse array configurations. This analysis is performed for the 3 × 3 MIMO case, and the results are shown in Figure 9.

The first benchmark is the three-element minimum redundancy array (MRA). As demonstrated in [23], the only MRA with N=3 elements is $z_{MRA}=\left[0,0.5,1.5\right]\lambda$. This configuration yields three unique baseline lengths (1, 2, and 3 in λ/2 units), thus maximizing the asymptotic angular resolution for a given number of elements. As shown by the ‘◊’ marker in Figure 9a, this array is considerably shorter than the array $z_{outlier-controlled}=\left[0\;1.67\;3.85\right]\lambda$ designed using our strategy (‘∆’ marker). Furthermore, we note that this MRA would not be admissible according to the selected minimum inter-element distance constraint (l ≈ 1.12λ), but we include it in the comparison for a comprehensive performance evaluation.

The second benchmark is an ambiguity-resistant NULA designed according to the criteria from [24]. This design strategy requires the two inter-element distances to be coprime multiples of λ/2. Among the many possible choices, we selected the configuration $z_{ambiguity-resistant}=\left[0\;1.5\;3.5\right]\lambda$ (‘□’ marker), which corresponds to coprime spacings of 3 and 4 units of λ/2. Notably, this array configuration falls within our generated admissible region and satisfies the imposed technological constraints.

Finally, we include a standard λ/2 ULA, with positions $z_{ULA}=[0,\;0.5,1]\lambda$ (‘○’ marker), as a conservative baseline. This configuration also violates our minimum distance constraint.

Figure 9b,c compare the MSE and maximum pairwise error probability for these configurations. As expected, the short-aperture ULA and MRA achieve asymptotic performance within the considered range of SNR values, exhibiting negligible outlier probability. However, their short baselines result in a relatively high CRB, which limits their asymptotic accuracy.

In contrast, the much sparser outlier-controlled and ambiguity-resistant NULAs achieve a significantly lower CRB, offering superior asymptotic performance. For these arrays, the outlier probability is no longer negligible in the threshold region, but satisfies the required constraint ($P_{m}<P_{max}=10^{-3},\;\forall m$) for SNR values larger than the design SNR of 15 dB.

The ambiguity-resistant design is inherently robust due to its coprime nature, but its maximum pairwise error probability is not explicitly controlled for a specific design SNR. In contrast, our strategy predicts and constrains $P_{max}$ to remain below the design threshold (indicated by the dashed line in Figure 9c) at the target SNR of 15 dB. This controlled robustness, combined with its marginally longer aperture, allows our proposed design to achieve both slightly better asymptotic accuracy and, more importantly, a significantly lower MSE in the critical threshold region.

In conclusion, this comparison highlights the practical value of our proposed strategy. Unlike fixed design criteria, our approach provides a flexible framework that defines an entire region of admissible arrays based on specific design parameters (SNR and $P_{max}$). This allows a designer to select the optimal configuration for their application, balancing performance trade-offs that are not captured by fixed-design arrays.

## 5. Conclusions

In this paper, we have extended the outlier-robust NULA design strategy introduced in [29] from SIMO to MIMO radar applications. Specifically, we studied how the number and positioning of transmitters impact both the design procedure and the DoA estimation accuracy. Through theoretical analysis, we have shown that MIMO operation not only enhances DoA estimation accuracy but also enhances array robustness to outliers. This flexibility enables a broader degree of flexibility when designing array configurations for AESA radar.

Moreover, we conducted a comparative analysis between the proposed MIMO-enabled NULA configurations and established NULA designs with inter-element distances at multiples of half wavelength. Our findings highlighted the effectiveness of our design strategy in identifying NULA configurations that achieve higher DoA estimation accuracy. This advantage holds even in scenarios where practical technological constraints make it impossible to resort to conventional design solutions.

The results of our analysis show that a three-element NULA array MIMO radar can be a viable and advantageous solution for low-cost sensors that require reliable and accurate DoA estimation but cannot afford either arrays with a large number of elements or large antenna apertures.

## Figures and Tables

**Table 1:** MSEs averaged within $U_{max}$ (in square radians).

Comparison	SNR	SIMO	2 × 3 MIMO Adjacent TX	2 × 3 MIMO External TX	3 × 3 MIMO
**Fixed array**	15 dB	$6.6\cdot 10^{-4}$	$1.4\cdot 10^{-4}$	$5.4\cdot 10^{-4}$	$0.9\cdot 10^{-4}$
	20 dB	$0.6\cdot 10^{-4}$	$0.4\cdot 10^{-4}$	$0.2\cdot 10^{-4}$	$0.3\cdot 10^{-4}$
**Fixed pairwise**	15 dB	$6.6\cdot 10^{-4}$	$3.0\cdot 10^{-4}$	$5.4\cdot 10^{-4}$	$0.3\cdot 10^{-4}$
	20 dB	$0.6\cdot 10^{-4}$	$0.2\cdot 10^{-4}$	$0.2\cdot 10^{-4}$	$0.1\cdot 10^{-4}$

**Table 2:** Ratio between MSEs averaged within $U_{max}$ between SIMO and MIMO.

Comparison	SNR	SIMO	2 × 3 MIMO Adjacent TX	2 × 3 MIMO Edge TX	3 × 3 MIMO
**Fixed array**	15 dB	1	4.6	1.2	7.3
	20 dB	1	1.3	2.5	2.0
**Fixed pairwise**	15dB	1	2.2	1.2	2.3
	20 dB	1	3.0	2.5	4.2

## Data Availability

The data presented in this study are available on request from the corresponding author.

## Appendix A. 3 × 3 MIMO

As discussed in Section 3, in the 3 × 3 MIMO case, the transmission and reception functionals are equal, since N=M=3, therefore, the likelihood function $L\left(\phi_{1},\phi_{2}\right)$ becomes:

(A1) $$L\left(\phi_{1},\phi_{2}\right)={\left|1+e^{j\phi_{1}}+e^{j\phi_{2}}\right|}^{4}={\left[3+2\cos\phi_{1}+2\cos\phi_{2}+2\cos\left(\phi_{2}-\phi_{1}\right)\right]}^{2}.$$

By using the 45° rotated axis system in Equation (15), we can rewrite Equation (A1) as:

(A2) $$L\left(x,y\right)={\left[1+4\cos y\cos x+4\cos^{2}x\right]}^{2}$$

By solving the equation $L\left(x,y\right)=L_{max}$ we obtain:

(A3) $$\cos y=\frac{\sqrt{L_{max}}-1}{4\cos x}-\cos x,$$

yielding:

(A4) $$y=\pm \mathrm{acos}\left\{\frac{\sqrt{L_{max}}-1}{4\cos x}-\cos x\right\}+2\pi m.$$

To obtain valid solutions, the argument of the arccosine must be limited as follows:

(A5) $$-1\leq \frac{\sqrt{L_{max}}-1}{4\cos x}-\cos x\leq 1.$$

This constraint is verified by the system of the two inequalities:

(A6) $$\left\{\begin{array}{c} \frac{4\cos^{2}x-4\cos x+1-\sqrt{L_{max}}}{\cos x}\leq 0\\ \frac{4\cos^{2}x+4\cos x+1-\sqrt{L_{max}}}{4\cos x}\geq 0 \end{array}\right..$$

Therefore, assuming $L_{max}\geq 1$, the first inequality is verified when:

(A7) $$\cos x\leq \frac{1-\sqrt[{4}]{L_{max}}}{2}\cup 0\leq \cos x\leq \frac{1+\sqrt[{4}]{L_{max}}}{2},$$

Similarly, the second inequality is verified when:

(A8) $$-\frac{1+\sqrt[{4}]{L_{max}}}{2}\leq \cos x\leq 0\cup \cos x\geq -\frac{1-\sqrt[{4}]{L_{max}}}{2}.$$

Calculating the union of these conditions gives:

(A9) $$-\frac{1+\sqrt[{4}]{L_{max}}}{2}\leq \cos x\leq \frac{1-\sqrt[{4}]{L_{max}}}{2}\cup -\frac{1-\sqrt[{4}]{L_{max}}}{2}\leq \cos x\leq \frac{1+\sqrt[{4}]{L_{max}}}{2},$$

which results in:

(A10) $$-\mathrm{acos}\left(\frac{-1+\sqrt[{4}]{L_{max}}}{2}\right)\leq x\leq \mathrm{acos}\left(\frac{-1+\sqrt[{4}]{L_{max}}}{2}\right).$$

Therefore, the locus of points in the $\left(\phi_{1},\phi_{2}\right)$ plane corresponding to the likelihood function cut at $L_{max}$ is obtained by applying the inverse coordinate transformation to Equation (37) and imposing condition Equation (A10), namely:

(A11) $$\left\{\begin{array}{c} \phi_{1}=\pm \mathrm{acos}\left\{\frac{\frac{L_{max}}{4\cos^{2}x}-1}{4\cos x}-\cos x\right\}+2\pi h-x\\ \phi_{2}=\pm \mathrm{acos}\left\{\frac{\frac{L_{max}}{4\cos^{2}x}-1}{4\cos x}-\cos x\right\}+2\pi k+x \end{array}\right.$$

within the limits of Equation (A10) for x.

## Appendix B. 2 × 3 MIMO

In the 2 × 3 MIMO case, with the phase reference chosen at the receiving-only array element, the transmission functional includes only the phase terms $\phi_{1}$ and $\phi_{2}$

(A12) $$\Psi_{2}\left(\phi_{1},\phi_{2}\right)={\left|e^{j\phi_{1}}+e^{j\phi_{2}}\right|}^{2}=2+2\cos\left(\phi_{2}-\phi_{1}\right),$$

while the reception functional is still the square root of Equation (A1) $\Psi_{3}\left(\phi_{1},\phi_{2}\right)$. Therefore, the likelihood function $L\left(\phi_{1},\phi_{2}\right)$ is:

(A13) $$L\left(\phi_{1},\phi_{2}\right)=2\left[1+\cos\left(\phi_{2}-\phi_{1}\right)\right]\left[3+2\cos\phi_{1}+2\cos\phi_{2}+2\cos\left(\phi_{2}-\phi_{1}\right)\right].$$

By using the 45° rotated axis system in Equation (15), we can rewrite Equation (A13) as:

(A14) $$L\left(x,y\right)=4\cos^{2}x\left(1+4\cos y\cos x+4\cos^{2}x\right)\leq L_{max}$$

The solution of the associated equation is:

(A15) $$\cos y=\frac{\frac{L_{max}}{4\cos^{2}x}-1}{4\cos x}-\cos x,$$

which yields

(A16) $$y=\pm \mathrm{acos}\left\{\frac{\frac{L_{max}}{4\cos^{2}x}-1}{4\cos x}-\cos x\right\}+2\pi m.$$

To obtain valid solutions, the following constraint must be imposed on the arccosine argument:

(A17) $$-1\leq \frac{\frac{L_{max}}{4\cos^{2}x}-1}{4\cos x}-\cos x\leq 1,$$

This constraint is verified by the system of the two inequalities:

(A18) $$\left\{\begin{array}{c} \frac{\cos^{4}x-\cos^{3}x+\frac{\cos^{2}x}{4}-\frac{L_{max}}{16}}{\cos^{3}x}\leq 0\\ \frac{\cos^{4}x+\cos^{3}x+\frac{\cos^{2}x}{4}-\frac{L_{max}}{16}}{\cos^{3}x}\geq 0 \end{array}\right..$$

The numerator of the first inequality has only two real roots:

(A19) $$R_{1,2}=\frac{1\pm \sqrt{1+4\sqrt{L_{max}}}}{4}\in R.$$

Therefore, assuming $L_{max}\geq 1$, the first inequality is negative if

(A20) $$\cos x\leq \frac{1-\sqrt{1+4\sqrt{L_{max}}}}{4}\cup 0\leq \cos x\leq \frac{1+\sqrt{1+4\sqrt{L_{max}}}}{4}.$$

Similarly, the numerator of the second inequality has only two real roots:

(A21) $$R_{3,4}=-\frac{1\pm \sqrt{1+4\sqrt{L_{max}}}}{4}\in R.$$

Therefore, assuming $L_{max}\geq 1$, the second inequality is positive if

(A22) $$-\frac{1+\sqrt{1+4\sqrt{L_{max}}}}{4}\leq \cos x\leq 0\cup \cos x\geq -\frac{1-\sqrt{1+4\sqrt{L_{max}}}}{4}.$$

The union of conditions Equations (A20) and (A22) yields:

(A23) $$-\frac{1+\sqrt{1+4\sqrt{L_{max}}}}{4}\leq \cos x\leq \frac{1-\sqrt{1+4\sqrt{L_{max}}}}{4}\;\cup \;-\frac{1-\sqrt{1+4\sqrt{L_{max}}}}{4}\leq \cos x\leq \frac{1+\sqrt{1+4\sqrt{L_{max}}}}{4},$$

which results in:

(A24) $$-\mathrm{acos}\left(\frac{-1+\sqrt{1+4\sqrt{L_{max}}}}{4}\right)\leq x\leq \mathrm{acos}\left(\frac{-1+\sqrt{1+4\sqrt{L_{max}}}}{4}\right).$$

Therefore, the locus of points in the $\left(\phi_{1},\phi_{2}\right)$ plane corresponding to the likelihood function cut at $L_{max}$ is obtained by applying the inverse coordinate transformation to equation Equation (A16) and imposing condition Equation (A24), namely:

(A25) $$\left\{\begin{array}{c} \phi_{1}=\pm \mathrm{acos}\left\{\frac{\frac{L_{max}}{4\cos^{2}x}-1}{4\cos x}-\cos x\right\}+2\pi h-x\\ \phi_{2}=\pm \mathrm{acos}\left\{\frac{\frac{L_{max}}{4\cos^{2}x}-1}{4\cos x}-\cos x\right\}+2\pi k+x \end{array}\right.,$$

within the limits of Equation (A24) for x.

(A26) $$-\mathrm{acos}\left(\frac{-1+\sqrt{1+4\sqrt{L_{max}}}}{4}\right)\leq x\leq \mathrm{acos}\left(\frac{-1+\sqrt{1+4\sqrt{L_{max}}}}{4}\right).$$
